# Differing determinants of disability trends among men and women aged 50 years and older

**DOI:** 10.1186/s12877-021-02574-3

**Published:** 2022-01-03

**Authors:** Ya-Mei Chen, Tung-Liang Chiang, Duan-Rung Chen, Yu-Kang Tu, Hsiao-Wei Yu, Wan-Yu Chiu

**Affiliations:** 1 Institute of Health Policy and Management, College of Public Health, National Taiwan University, Room 633, No. 17, Xu-Zhou Road, Taipei, 100 Taiwan; 2 Institute of Health Behaviors and Community Sciences, College of Public Health, National Taiwan University, Room 636, No. 17, Xu-Zhou Road, Taipei, 100 Taiwan; 3 Institute of Epidemiology and Preventive Medicine, College of Public Health, National Taiwan University, Room 539, No. 17, Xu-Zhou Road, Taipei, 100 Taiwan; 4 Department of Gerontology and Health Care Management, Chang Gung University of Science and Technology, Room 1406, No. 261, Wenhua 1st Rd, Taoyuan, 333 Taiwan

**Keywords:** Disability trajectory, Sex, Leisure-time activities, Determinants

## Abstract

**Background:**

Researchers have emphasized the importance of examining how different factors affect men’s and women’s functional status over time. To date, the literature is unclear about whether sex affects the rate of change in disability in middle to older age. Researchers have further emphasized the importance of examining how different factors affect men’s and women’s functional status over time. We examined (a) sex differences in disability trends and (b) the determinants of the rate of change in disability for men and women 50 years and older.

**Methods:**

This study utilized the Taiwan Longitudinal Study on Aging Survey, a nationally representative database (four waves of survey data 1996–2007, *N* = 3429). We modeled and compared the differences in disability trends and the influences of determinants on trends among men and women using multiple-indicator and multiple-group latent growth curves modeling (LGCM). Equality constraints were imposed on 10 determinants across groups.

**Results:**

Once disability began, women progressed toward greater disability 18% faster than men. Greater age added about 1.2 times the burden to the rate of change in disability for women than men (*p* < 0.001). More comorbidities also added significantly more burden to baseline disability and rate of change in disability among women than men (*p* < 0.001), but women benefited more from higher education levels in lower baseline disability and slower rate of change. Having a better social network was associated with lower baseline disability among women only (*p* < 0.05). For both men and women, physically active leisure-time activities were beneficial in lower baseline disability (*p*_men and women_ < 0.001) and rate of change in disability (*p*_men_ < 0.01; *p*_women_ < 0.05), with no significant differences between groups.

**Conclusions:**

Age may widen the sex gap in the rate of change in disability. However, both sexes benefit from participating in leisure-time activities. Promoting health literacy improves health outcomes and physical function among women.

**Supplementary Information:**

The online version contains supplementary material available at 10.1186/s12877-021-02574-3.

## Background

Maintaining physical function has been a key public health priority for many fast-aging societies for some time. Over the past 10 years, researchers’ attention has been drawn to identifying factors associated with changes in physical function trends [10, 11, 16, 50]. Sex differences in the nature and range of health pathways over the life course are among these factors, and there have been calls to further delineate sex patterns and health-related consequences [33].

Studies have now shown that sex differences in functional status among older adults reflect not only biological differences but also differences in privilege and power based on sex identity and past decision making [13, 15, 27, 33, 39, 51]. Researchers have further emphasized the importance of examining how different factors affect men’s and women’s functional status over time [10, 27, 57, 58]. When Liang et al. [26] examined functional changes over time among middle-aged and older men and women from a life course perspective, they found that decreases in functional status were more accelerated—in terms of both baseline disability and rate of change in disabilities—among women than men. Chen et al. [10] reported that sex may not be a risk factor for developing initial disability, yet women who do develop disability may be at greater risk than men of faster increases in disability. However, how much faster women’s rate of change in disability may be remains unclear.

Latent growth curves modeling (LGCM) has been e recently advocated as a better method for addressing questions related to individual change over time because it provides estimates of an individual growth curve for each subject, including estimated baseline values and rates of change, while also taking individual variations into consideration [35, 36, 43]. In addition, LGCM gives researchers more flexibility to estimate patterns of change for its ability to establish nonlinear growth trajectories [14, 36].

Only a few determinants have yet been examined for their association with older adults’ disability trends. These determinants have included both mutable determinants, such as health behaviors and social support, and immutable determinants, such as age and number of comorbidities [2, 9, 10, 27, 48, 54]. However, the current literature remains unclear as to what extent these determinants affect disability trends among men and women, and especially how they affect rate of change in disability [47, 56]. Thus, our study aimed to examine both (1) sex differences in disability trends and (2) the different determinants of the rate of change in disability for men and women in middle age and older.

## Methods

### Data and sample

**Fig. 1:**
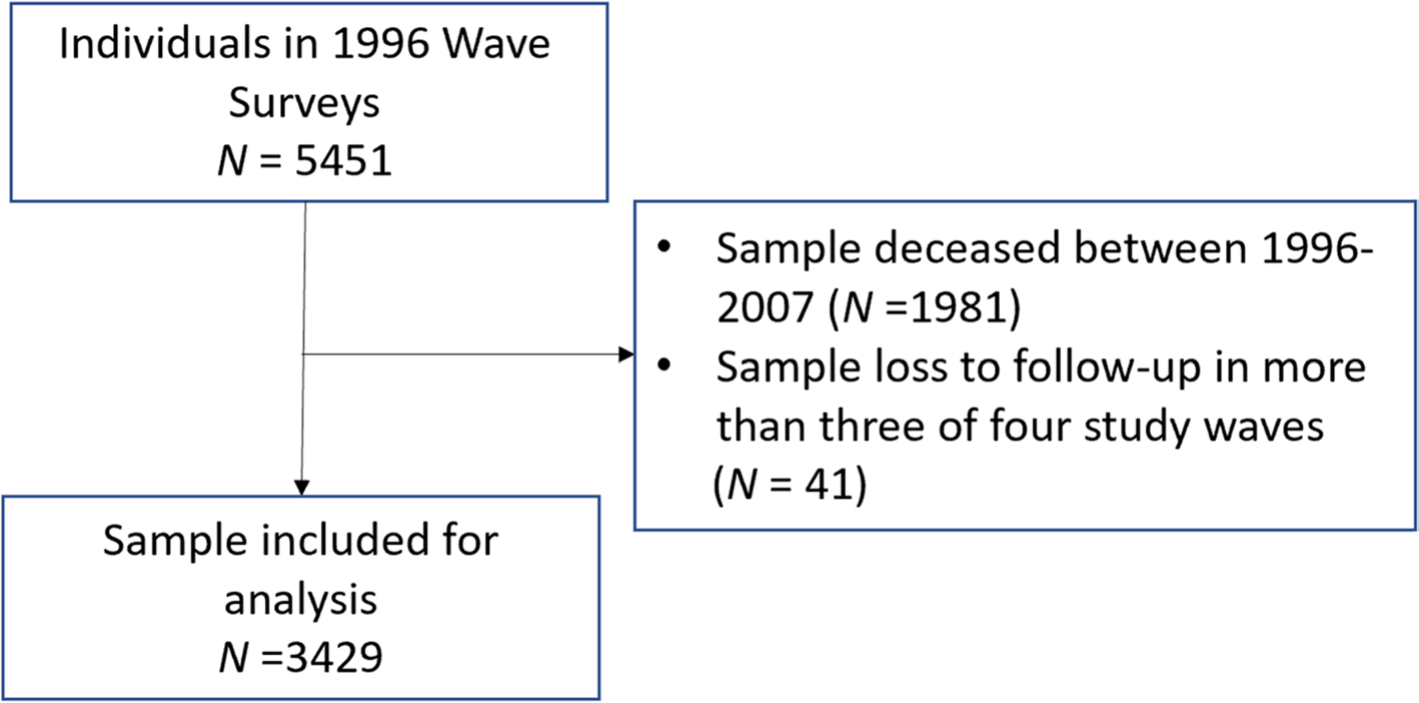
Flow Diagram of the Taiwan Longitudinal Study on Aging Cohort Sample and Follow-Up Surveys

This study used the Taiwan Longitudinal Study on Aging (TLSA), which was a national population-representative survey launched in 1989, aged 50 and up, and followed up in 1993, 1996, 1999, 2003, and 2007. It was conducted by the Taiwan Provincial Institute of Family Planning (which later became the Bureau of Health Promotion of the Taiwan Department of Health) and the University of Michigan, with support from Taiwan’s government and the U.S. National Institute on Aging (Taiwan Provincial Institute of Family Planning et al., 1989). A second cohort, aged 50–67 years, was added in 1996 and followed in the subsequent waves. Data quality and details of the survey have been presented previously [10, 27, 56]. We included four waves of survey data—from the 1996 to 2007 surveys—in this study’s analysis, due to certain key variables are available only from the data collected in the 1996–2007 surveys. This study included 3429 people who survived to the 2007 survey and had completed at least one of the four surveys for analysis (please see Fig. 1 for details). All subjects provided written informed consent, and the ethical committee of the Bureau of Health Promotion, Taiwan, approved the national survey study.

Respondents were asked to choose between two options for sex: *Male* or *Female.* Sample weights representing Taiwan’s population aged 50 and older as of 1996 were included. Missing values were replaced using the multiple imputation procedure in Mplus 7.3 [25].

### Measures

#### Disability trends

**Fig. 2:**
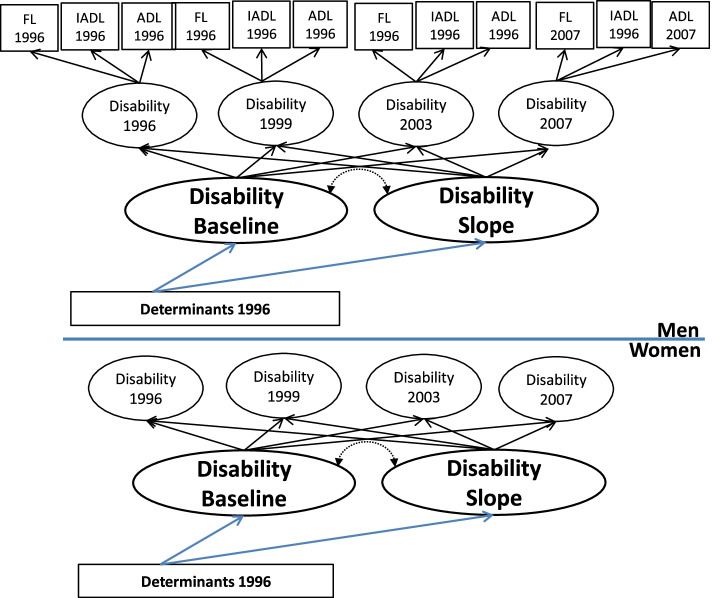
Multiple-Group Latent Growth Curve Model for Disability and Disablement Factors Among Men and Women 50 Years and Older. Notes: FLxxxx = Nagi’s functional limitation in xxxx (year); IADLxxxx = instrumental activities of daily living in xxxx (year); ADLxxxx = activitiesof daily living in xxxx (year); GFDxxxx = general functional disability in xxxx (year). The indicators for each latent disability variable were illustrated for both men and women

In this study, we applied multiple-indicator latent growth curve modeling (LGCM), and included a latent variable for disability trends assessed by three indicators—activities of daily living (ADLs [22];), instrumental activities of daily living (IADLs [21];), and Nagi’s functional limitations [37]. These three indicators were all measured at 1996, 1999, 2003, and 2007 four time points (Disability 1996 to Disability 2007; please see Fig. 2 for illustration).

Using multiple functional outcome measures to assess functional limitations in the older population has been recommended in the literature [10, 23, 57]. The National Research Council has suggested including functional limitations in addition to ADL and IADL limitations to better enable researchers to understand the disability process [38]. LGCM allows researchers to include multiple indicators to estimate the growth curve of the general process of functional disability and the advantages of using multiple-indicator LCGM has been addressed in previous studies [4, 10, 18, 44].

Details regarding the interview contents of TLSA data have been presented previously [10, 11]. The three indicators we included—Nagi’s functional limitations, ADL disability, and IADL disability—assess physical function from multiple perspectives [44]. The severity level for each activity in these three indicators was assessed with four grades, from 0 (*no limitation*) to 3 (*unable to do*). The severity level for each category was then summed (see Table 1).

**Table 1:** Characteristics of the sample (*N* = 3249)

	Min	Max	Men (*N* = 1718)		Women (*N* = 1711)		*P*-value
			Mean	*SD*	Mean	*SD*	
Determinants (1996)							
Age	50	96	63.960	8.113	63.880	8.409	0.778
Education	0	17	6.950	4.609	3.200	3.897	< 0.001
Comorbidities	1	5	3.530	1.043	3.130	1.043	< 0.001
Depression	0	30	4.190	4.689	5.990	5.787	< 0.001
No alcohol consumption	0	1	.630	.484	.930	.252	< 0.001
Leisure-time activities, recreational	0	5	2.900	1.0200	2.170	0.947	< 0.001
Leisure-time activities, physically active	0	4	1.300	1.045	1.150	1.000	< 0.001
Social network	0	176	20.060	17.727	18.200	15.928	< 0.001
Social support	4	20	16.270	2.909	16.21	2.882	0.515
Use of assistive devices	0	3	1.280	0.728	1.250	0.707	0.153
FL1996	0	24	0.83	2.541	2.18	3.713	< 0.001
FL1999	0	24	1.42	3.115	3.52	4.712	< 0.001
FL2003	0	24	2.51	4.413	5.32	5.93	< 0.001
FL2007	0	24	4.01	6.191	6.99	7.024	< 0.001
ADL1996	0	18	0.08	0.941	0.12	0.997	0.200
ADL1999	0	18	0.13	1.051	0.23	1.333	0.010
ADL2003	0	18	0.34	1.873	0.72	2.719	< 0.001
ADL2007	0	18	1.19	3.795	1.9	4.589	< 0.001
IADL1996	0	18	0.42	1.657	1.22	2.671	< 0.001
IADL1999	0	18	0.55	1.826	1.67	3.252	< 0.001
IADL2003	0	18	1.24	3.102	2.87	4.567	< 0.001
IADL2007	0	18	2.62	4.944	4.43	5.766	< 0.001

*Note*. *p < 0.05, **p < 0.01, ***p < 0.001. *FLxxxx* Nagi’s functional limitation in xxxx (year), *IADLxxxx* Instrumental activities of daily living in xxxx (year), *ADLxxxx* Activities of daily living in xxxx (year)

#### Factors that influence disability trends by sex

Our analysis examined 10 determinants—age, education level, number of comorbidities, depression, alcohol consumption (yes or no), recreational and physically active leisure-time activities, social network, social relations, and use of assistive devices—that have been reported in earlier studies to influence older adults’ disability trends [10]. Data on these factors were drawn from the baseline TLSA survey (the 1996 survey).

Age and education level were measured by the actual year of age and education received. Comorbidities were measured as number of reported chronic health conditions (e.g., hypertension, diabetes mellitus, heart disease, stroke, cancer, pulmonary disease, arthritis, gastric ulcer, liver disease, hip fracture, cataract, renal disease, gout, and spinal spurs). Depression was assessed by the 10-item version of the Center for Epidemiologic Studies Depression Scale (CES-D), which represents levels of depressive symptoms ranging from 0 to 30 [42]. Presence or absence of alcohol consumption was assessed by a question about drinking habits.

Leisure-time activities include the following: (1) watching television, (2) listening to music or radio, (3) reading, (4) playing mahjongg or chess, (5) gathering with friends or family, (6) gardening, (7) taking a walk, (8) outdoor activities such as tai chi, and (9) group activities. Factor loadings ranged from .500 to .863 [8]. Based on information from previous studies, we grouped the first five activities into recreational leisure-time activities and the latter four activities into physically active leisure-time activities [1, 8, 19, 55]. Please see Supplementary Table 1 for detailed information about our categorization of leisure-time activities.

Social network was assessed by frequency of contact with relatives and friends per week [59]. Social support was assessed with four items measuring level of satisfaction (1–5) with emotional support, resulting in a sum score ranging from 4 to 20 (higher scores represent greater satisfaction with support). The four items were “Someone listens to me,” “Someone cares about me,” “My family cares about me” (level of satisfaction), and “Someone will take care of me if I become ill.” The internal consistency was 0.822. and the factor loading ranged from .733 to .800. Use of assistive devices was assessed by individual’s use of four types of devices (0–4): glasses, hearing aids, dentures, and wheelchairs, resulting a sum score ranging from 0 to 4.

### Latent growth curve modeling and analysis

We used both multiple-indicator and multiple-group LGCM to test the different influences of the determinants for both groups (men and women) and applied testing for partial invariance [6]. We applied the second-order growth model [30] with the assumption that all indicators shared the same trait and the same trait growth process, and all indicators shared the same state residual component within scaling differences [4]. Figure 2 shows the setup of our multiple-group LGCM. Each latent variable of disability was identified by three physical function measures: ADLs, IADLs, and functional limitations. The upper half and lower half of Fig. 2 indicate the baseline and rate of change in disability, which indicates speed of progression toward disability in each group. This growth process contains two latent factors of baseline disability and two latent factors of disability slope (i.e., rate of change in disability per year from 1996 to 2007) over the 11 years of the study period, for men and women respectively. Baseline and rate of change in disability were thus measured by four latent variables, Disability 1996 to Disability 2007. The determinants were included to assess the impact on disability baselines and slopes across sex. The measurement errors were set to be correlated.

Our analysis was based on comparisons of different models in which parameters were constrained or not constrained to be equal. The analysis procedure was multi-stepped and included (1) testing unconditional multiple-group nonlinear and linear growth models to disability trends among men and women and comparing the model fit; (2) testing unconstrained models allowing all parameters to be freely estimated across groups; (3) testing constrained models assuming that parameters are equal across groups, and comparing by using chi-square difference tests between fully constrained and unconstrained models; and (4) comparing structural parameters by systematically constraining and unconstraining specific paths to determine which paths contribute to significant differences between the two. Equality constraints were imposed on the 10 determinants assessed across groups [40, 43].

In this study, the LGCM was fit to data using Mplus (version 7.1) with a robust maximum likelihood estimator. Four model fit indexes were applied to evaluate the adequacy of model fit [24]: (a) chi-square statistics [20], (b) the Bentler Comparative Fit Index (i.e., CFI ≥ 0.9 [3, 5];, and (c) root mean square error of approximation (i.e., RMSEA ≤0.05) with 90% confidence interval [45]. Significant chi-square difference (∆χ^2^) tests, which were used to determine significant differences between constrained and unconstrained models, indicated determinants that showed significantly different influences on men’s and women’s disability trends [36].

## Results

### Sample characteristics

The sample was about 50% women, with a mean age in 1996 of 63.96 (*SD* = 8.113) years for men and 63.88 (*SD* = 8.409) years for women. Detailed information regarding the sample included for analysis is presented in Table 1, which also shows that the level of disability among men and women continually increased over time.

Both men and women started out with less severe disabilities in 1996, with grades of 0.83 (*SD* = 2.54) and 2.18 (*SD* = 3.71) for Nagi’s functional limitations, 0.42 (*SD* = 1.66) and 1.22 (*SD* = 2.67) for IADLs, and 0.08 (*SD* = 0.94) and 0.12 (*SD* = 1.00) for ADLs. Baseline grades for men and women were significantly different for Nagi’s functional limitations (*p* < 0.001) and IADLs (*p* < 0.001). Functional disabilities among these groups increased over the years; in 2007, more severe disability was measured in Nagi’s functional limitations (men: 1.04, *SD* = 6.19 vs. women: 6.99, *SD* = 7.04), IADLs (2.62, *SD* = 4.94; 4.43, *SD* = 5.77), and ADLs (1.19, *SD* = 3.80; 1.9, *SD* = 4.59).

### Latent growth curve model

The unconditional modeling results showed that the nonlinear models fit better to each group’s disability trends (χ2 [66, *N* = 3429] = 768.275 [men 303.501 vs. women 464.774], *p* < 0.001; CFI = .941; RMSEA = .058). The multiple-group model showed that disabilities increased more slowly among women than men at Wave 3 of the survey, but increased at a faster rate among women at the Wave 4 survey. Baseline disability levels and rate of change in disability were constrained in separate models and compared to the unconditional and unconstrained model. The baseline disability levels showed no significant difference between the two groups, but once disability began, the progression toward greater disability was almost 18% faster among women than men (B: 0.694 men vs. 0.817 women; *p* < 0.01). The detailed results of the nonlinear LGCM for disability are presented in Supplementary Table 2 and Supplementary Fig. 1.

### Factors that influence men and Women’s rate of change in disability differently

The conditional nonlinear LGCM also fit well to the disability trends (χ2 [303, *N* = 3429] = 1824.20 [745.343 men vs. 1078.857 women], *p* < 0.001; CFI = .928; RMSEA = .054). Among women compared to men, greater age added 1.23 times the burden to the rate of change in disability (β_Age slope_: 0.380, *p* < 0.001 men vs. 0.467, *p* < 0.01 women, ∆χ2 = 11.997, *p* < 0.001). Higher education level was associated with lower rate of change in disability for women but not men, although the differential impact between the two groups was only marginally significant (β _Education slope_: −0.061, *p* > 0.05 men vs. -0.066, *p* < 0.01 women, ∆χ^2^ = 3.623, *p* = 0.057). Number of comorbidities was found to add burden to the rate of change in disability in both groups, but the impact of not significantly different between groups (β _Comorbidities slope_: 0.108, *p* < 0.01 men vs. 0.146, *p* < 0.001 women, ∆χ = 2.236, *p* > 0.05). Finally, both men and women benefited from the effect of physically active leisure-time activity on slowing the rate of change toward greater disability. Although the differential impact between the two groups on the rate of change was again only marginally significant, men tended to benefit more than women from physically active leisure-time activities (β _Physically active LTA slope_: −0.092, *p* < 0.01 men vs. -0.063, *p* < 0.05 women, ∆χ^2^ = 3.672, *p* = 0.055).

Other factors studied, such as depression, alcohol habits, and having better social networks, showed differential impacts between the two groups only on baseline disabilities and not the rate of change in disability. Please see Table 2 for details.

**Table 2:** Differential impacts of determinants on men’s and women’s disability trends (*N* = 3429)

Determinants	Intercept (Baseline)			Slope (Rate of Change)		
	Men	Women	Χ^2^diff test	Men	Women	Χ^2^diff test
	Estimate β (SE) Standardize β	Estimate β (SE) Standardize β		Estimateβ (SE) Standardize β	Estimateβ (SE) Standardize β	
Age	0.021 (0.008) 0.100*	0.083 (0.008) 0.265***	22.227***	0.017 (0.002) 0.380***	0.026 (0.002) 0.467***	11.997***
Education	0.003 (0.013) 0.009	−0.033 (0.012) -0.055**	5.689*	−0.004 (0.002) -0.061	−0.007 (0.003) -0.066**	3.632 (0.057)^a^
Comorbidities	0.101 (0.036) 0.090**	0.242 (0.045) 0.157***	6.686*	0.026 (0.009) 0.108**	0.041 (0.009) 0.146***	2.263
Depression	0.073 (0.017) 0.220***	0.101 (0.017) 0.240***	−0.990	−0.001 (0.002) -0.013	−0.001 (0.002)-0.015	2.535
No alcohol consumption	0.271 (0.064) 0.086***	0.09 (0.14) 0.024	4.785**	−0.005 (0.019) -0.007	−0.034 (0.041) -0.020	3.444
Leisure-time activities, recreational	−0.163 (0.067) -0.109**	−0.127 (0.054) -0.069**	2.527	−0.004 (0.009) -0.012	−0.001 (0.012) -0.002	3.728
Leisure-time activities, physically active	−0.136 (0.04) -0.094***	−0.291 (0.06) -0.125***	4.970*	−0.029 (0.008) -0.092***	−0.026 (0.011) -0.063*	3.672 (0.055)^a^
Social network	−0.001 (0.002) -0.013	−0.005 (0.003) -0.037*	4.510*	0.00 (0.001) −0.004	0.000 (0.001) -0.007	3.178
Social support	0.023 (0.015) 0.043	0.036 (0.025) 0.043	2.490	-0.004 (0.003) -0.033	−0.003 (0.005) -0.021	3.021
Use of assistive devices	0.149 (0.064) 0.071**	0.108 (0.099) 0.032	2.123	−0.016 (0.015) -0.035	−0.02 (0.016) -0.033	2.979
Model fit	χ2 [303, *N* = 3429] = 1824.20 [Men: 745.343 vs. Women: 1078.857], *p* < 0.001; CFI = .928; RMSEA = .054					

*Note*. *p < 0.05, **p < 0.01, ***p < 0.001

^a^ Marginally significant *p*-values of Χ^2^diff test (*p* < 0.1) are presented when at least one of the estimates for men or women were significant

## Discussion

Past studies have returned inconsistent results on whether sex is associated with different levels of burden on disability trends among middle-aged and older adults [10, 26, 27, 46, 56]. Our study findings advance this body of knowledge by confirming that while middle-aged and older men and women demonstrate no differences in baseline disability, once disability has begun, the rate of change in disability is faster among women than men—18% faster in our study. However, it is necessary to be cautious when interpreting difference in rate of change between groups. In this case, since both men’s and women’s trends progressed in a curved manner, the differences in rate of change may also be different across time.

Another key contribution from this research lies in its focus on how mutable and immutable determinants associate with disability trends differently by sex. Age posed greater risks to disability progression among women than men, while women received marginally more benefit than men from education. However, both women and men benefited from engaging with physically active leisure-time activities through a slower progression in disability.

### Age adds more burden for women than men

Age and comorbidities are known to be significant factors for disability ([12, 13, 15, 27, 29, 46, 54]). Most determinants identified in past studies [17, 32, 47, 48, 54, 56] showed significantly different influences by sex only on baseline disabilities in the current study. Age was the only determinant that our study showed to have different influences on change in disability among middle-aged and older men and women. We found that age added 1.23 times the burden in rate of change in disability on women. This indicates that age may also widen the existing gap between men and women in the rate of change in disability.

Some may argue that women have longer life expectancies, and therefore may experience faster increases in disability simply due to their older age. However, in our analysis, the mean age of the groups of men and women in all four waves of data was not significantly different (see Supplementary Table 1). In addition, past studies have suggested that if chronic illness is well controlled, aging is not inevitably related to functional decline [10, 46].

Our study further indicates that while age adds more burden to women than to men in terms of rate of change in disability, comorbidities add burden to both groups. Our results showed that even with age controlled in the model, number of chronic illnesses still added as much as two times the burden to women’s baseline disability as to men’s baseline disability. However, the number of chronic illnesses added burden to rate of change in disability equally for men and women. Thus, preventing the development of chronic illness and decreasing the numbers of chronic illnesses should be the first priority for maintaining physical function for both men and women. Preventing disability during aging, especially for women, should be a focus in future policy-making [12].

The influence of age on the overall disability trend may also be different between adults in middle age and older. An earlier study [56] that used the same dataset as our study also examined trends among adults age 50 years and older. That study found that those who were 50 to 59 years old at baseline showed similar patterns of disability trends as those in other age ranges, but had different probabilities of entering into different disability trend patterns [56]. Yu et al.[54] also indicated that those who are younger are more likely to enter a heathier trend. Careful attention and explanation of participants’ age ranges is necessary, and further studies are recommended to examine the influence of age on disability trends between middle-aged and older adults.

### Higher education may benefit women but not men

Past studies have shown that a higher education can be a protective factor against developing disability in later life. More-educated older adults invest in late-life health through healthier behaviors and are thus at less risk of developing and increasing functional limitations or physical disabilities [11, 29]. However, the role of education on disability for men versus women has been controversial in the literature. In our study, women with higher education levels not only had lower baseline disability but also tended to show slower progression toward greater disability. No beneficial effects of education were observed among men, on baseline or progression toward disability.

These findings are not consistent with those of past studies. Zimmer et al. [56] pointed out the possibility of an intertwined influence between sex and education on older adults’ disabilities, noting that education seems to be less important to predicting disability trajectory among women than it is among men, and that women with less education than their husbands may benefit in part from influences tied to the husbands’ characteristics. In contrast, we found education had a beneficial effect on disability only for women and not for men, though the difference was only marginally significant. Other past studies have emphasized that women’s health behaviors are associated with their levels of education and health literacy [28]. Women with higher education may particularly benefit from such characteristics and therefore benefit from lower baseline and slower progression toward disabilities [11].

Based on our study findings, then, continuing to promote higher education levels for women in Taiwan should be considered in future health policy-making. Past studies have also suggested that promoting health literacy among women promotes better health outcomes and physical function, so this could also be considered a policy priority [11, 31, 52].

As to why men did not benefit from higher education in this study, a review study has pointed out that men responded better toward male-specific health-related information, rather than assuming all health education efforts are equally effective with everyone [41]. Planning different health education campaigns for women and men is recommended.

### Leisure-time activities benefit both women and men

Many past studies have reported that being physically active reduces disability in older adults and prevents new-onset ADL disabilities [17, 47, 48, 54, 56]. Strobl et al. [47] suggested that men benefit more than women from physically active leisure-time activities in terms of developing late-life disability, but that once disability begins, there appears to be no further association with the severity of disability. Our findings were thus partly in line with the results of previous studies [11, 47].

Our study findings indicated that once disability began, physically active leisure-time activities were strongly associated with slower progress toward severe disability among both men and women. Our study further showed that men seemed to benefit more than women from physically active leisure-time activities in terms of slower progression toward disability. Past studies have suggested that sex differences might contribute to different outcomes from physical activities, such as non-fatal chronic conditions, lower muscle strength, and lower bone density in women [39].. Past studies have also shown that men and women age 50 and older prefer different physical activities [34, 49] and that the percentage of women reporting high levels of physical activity was significantly lower than the percentage of men reporting high activity levels [7, 47]. These may also lead to fewer disadvantages in making slower progression in disabilities among women. Further research is needed to understand to what extent the level of physical activity affects middle-aged and older men’s and women’s rate of change in disability.

Strobl et al. [47] have pointed out that their study sample cannot be representative of all older people, particularly those who do not choose to participate in research due to disabling conditions. Our study was based on a representative survey of the population, which included people who had and had not participated in leisure-time activities. Different target samples might also contribute different findings from the current study and past studies [47]. However, promoting physically active leisure-time activities for both sexes is a promising strategy.

### Limitations

Several limitations need to be addressed. The first is that for parsimony of the model, we investigated only 10 of the commonly studied determinants of sex disparities. A number of other variables known to influence the development of disability (e.g., cognitive impairments and economic status) were not included due to data availability. The current study can still serve as a foundation for further studies that examine a more comprehensive set of determinants and their associations with different functional outcomes in men and women. The second limitation is that, as with many longitudinal studies, this study had selective attrition. We included in the analysis only those men and women who survived the 11-year period from 1996 to 2007.

In addition, although differences in mean age of the men and women included in our analysis remained nonsignificant across all four waves of data, those who were not included were more likely to be older and have more severe disabilities. Thus, our results shall be interpreted with caution.

The advantage of LGCM is in examining the distribution of trajectories that vary continuously across individuals [57]. The disadvantage is that including deceased individuals may lead to sampling error and bias the estimation of the disability trajectory, particularly for those who experience early onset of disability [56]. As a result, LGCM tends to favor separate estimates for surviving and deceased respondents [53], and we decided not to include the deceased in our analyses. However, this may limit our ability to generalize our findings to those who died, and our results should thus be interpreted with caution.

## Conclusions

To date, very few population-based studies have aimed to understand the issue of sex-specific differences in the impact of determinants on the rate of change in disability among men and women in middle aged and older. We found that while women did not bear a larger burden of baseline disability than men, once disability began, women’s progression toward greater disability occurred faster. Only age had a different impact by sex on the rate of change in disability; while education and physically active leisure-time activities marginally benefited both women and men through slower progression toward disabilities. Physically active leisure-time activities are mutable determinants that promise to be beneficial for both sexes, though men seemed to benefit more than women from participating in these activities. Promoting physically active leisure-time activities should be a priority for future policy and interventions aimed at maintaining adults’ physical functioning over time—for both men and women. Better control of chronic illness, preventing disability at earlier ages, and promoting middle-aged and older women’s education also remain important policy goals.

## Supplementary Information

**Additional file 1: Supplementary Table 1**. Detailed Descriptions of the Measures (*N* = 3249). **Supplementary Table 2**. Descriptive Results and Factor Loadings of Nonlinear Unconditional LGCM for Disability Trends in Four Waves of Survey Data (N = 3429).

**Additional file 2: Supplementary Figure 1**. Disability Trends of Men and Women Over 11 years of study period.

## Data Availability

The data that support the findings of this study are available from Taiwan’s Health and Welfare Data Science Center but restrictions apply to the availability of these data, which were used under license for the current study, and so are not publicly available. Data are however available from the authors upon reasonable request and with permission of Taiwan’s Health and Welfare Data Science Center. Further contact information is available through the following URL: https://dep.mohw.gov.tw/dos/cp-5119-59201-113.html.

## Abbreviations

FL: Functional limitation
IADL: Instrumental activities of daily living
ADL: Activities of daily living
