# Supplementary material for: Differing determinants of disability trends among men and women aged 50 years and older
Source: BMC Geriatr. 2022 Jan 3;22:11. doi: 10.1186/s12877-021-02574-3 (PMC8722081; doi:10.1186/s12877-021-02574-3)
Supplement: Supplementary file 1 — Additional file 1: Supplementary Table 1. Detailed Descriptions of the Measures (N = 3249). Supplementary Table 2. Descriptive Results and Factor Loadings of Nonlinear Unconditional LGCM for Disability Trends in Four Waves of Survey Data (N = 3429). [file 12877_2021_2574_MOESM1_ESM.docx]

Supplementary Table 1.

Detailed Descriptions of the Measures (*N* = 3,249)

|  |  |  | Men (N = 1,718) | | Women (N = 1,711) | | |  |
| --- | --- | --- | --- | --- | --- | --- | --- | --- |
|  | Min | Max | Mean | SD | Mean | SD | | p-value |
| Age 1996 | 50 | 96 | 63.96 | 8.113 | 63.88 | 8.409 | | 0.778 |
| Age 1999 | 53 | 99 | 66.96 | 8.113 | 66.88 | 8.409 | | 0.778 |
| Age 2003 | 57 | 102 | 70.29 | 8.115 | 70.21 | 8.386 | | 0.770 |
| Age 2007 | 61 | 106 | 74.34 | 8.145 | 74.27 | 8.417 | | 0.806 |
| Determinants (1996) |  |  |  |  |  |  | |  |
| Recreational leisure-time activities |  |  |  |  |  | |  |  |
| Watching television | 0 | 1 | 0.98 | 0.153 | 0.96 | | 0.194 | 0.010 |
| Listening to music or radio | 0 | 1 | 0.43 | 0.495 | 0.35 | | 0.478 | <0.001 |
| Reading | 0 | 1 | 0.66 | 0.475 | 0.23 | | 0.422 | <0.001 |
| Playing mahjongg or chess | 0 | 1 | 0.16 | 0.365 | 0.05 | | 0.217 | <0.001 |
| Gathering with friends or family | 0 | 1 | 0.68 | 0.467 | 0.58 | | 0.494 | <0.001 |
| Physically active leisure-time activities |  |  |  |  |  | |  |  |
| Gardening | 0 | 1 | 0.32 | 0.467 | 0.34 | | 0.474 | 0.226 |
| Taking a walk | 0 | 1 | 0.60 | 0.491 | 0.55 | | 0.498 | 0.004 |
| Outdoor activities | 0 | 1 | 0.29 | 0.455 | 0.15 | | 0.359 | <0.001 |
| Group activities | 0 | 1 | 0.08 | 0.275 | 0.10 | | 0.300 | 0.069 |
| Social network |  |  |  |  |  | |  |  |
| Number of siblings | 0 | 15 | 6.2311 | 3.82960 | 5.3553 | | 4.01299 | <0.001 |
| Number of grandchildren | 0 | 30 | 1.5611 | 2.55984 | 2.4354 | | 3.21151 | <0.001 |
| Number of relatives | 0 | 77 | 4.8847 | 8.51889 | 4.1502 | | 7.05618 | 0.006 |
| Number of neighbors or friends | 0 | 77 | 7.4051 | 9.52109 | 6.2782 | | 8.80688 | <0.001 |
| Social support |  |  |  |  |  | |  |  |
| Someone listens to me | 1 | 5 | 3.80 | 1.061 | 3.84 | | 1.033 | 0.322 |
| Someone cares about me | 1 | 5 | 4.24 | 0.807 | 4.19 | | 0.817 | 0.043 |
| Family cares about me | 1 | 5 | 4.11 | 0.766 | 4.07 | | 0.803 | 0.097 |
| Someone will take care of me if I become ill | 1 | 5 | 4.12 | 0.951 | 4.12 | | 0.882 | 0.958 |

*Note*. **p* < 0.05, ***p* < 0.01, ****p* < 0.001.

Supplementary Table 2.

Descriptive Results and Factor Loadings of Nonlinear Unconditional LGCM for Disability Trends in Four Waves of Survey Data (N = 3,429).

*Note*. **p* < 0.05, ***p* < 0.01, ****p* < 0.001.

|  |  | Factor Loadings (Standardized) | | | | | |
| --- | --- | --- | --- | --- | --- | --- | --- |
|  |  | Men | | | Women | | |
|  |  | Intercept | Slope |  | | Intercept | Slope |
| 1996 |  | 0.773 (0.077) | 0.000 (0.000) |  | | 0.978 (0.033) | 0.00 (0.00) |
| 1999 |  | 0.706 (0.071) | 0.235(0.037) |  | | 0.764 (0.044) | 0.213(0.037) |
| 2003 |  | 0.434 (0.063) | 0.752(0.035) |  | | 0.474 (0.034) | 0.665(0.035) |
| 2007 |  | 0.272 (0.042) | 0.590(0.035) |  | | 0.361 (0.028) | 0.639(0.031) |
| Mean |  | 0.000 (0.000) | 0.694(0.041) |  | | 0.359 (0.024) | 0.817(0.033) |
|  |  | χ2 [103, *N* = 3,429] = 646.219 [Male: 230.899 vs Female 415.393], *p* < .001; CFI = .951; RMSEA = .055 | | | | | |
