# Supplementary figures and images for: Differing determinants of disability trends among men and women aged 50 years and older
Source: BMC Geriatr. 2022 Jan 3;22:11. doi: 10.1186/s12877-021-02574-3 (PMC8722081; doi:10.1186/s12877-021-02574-3)

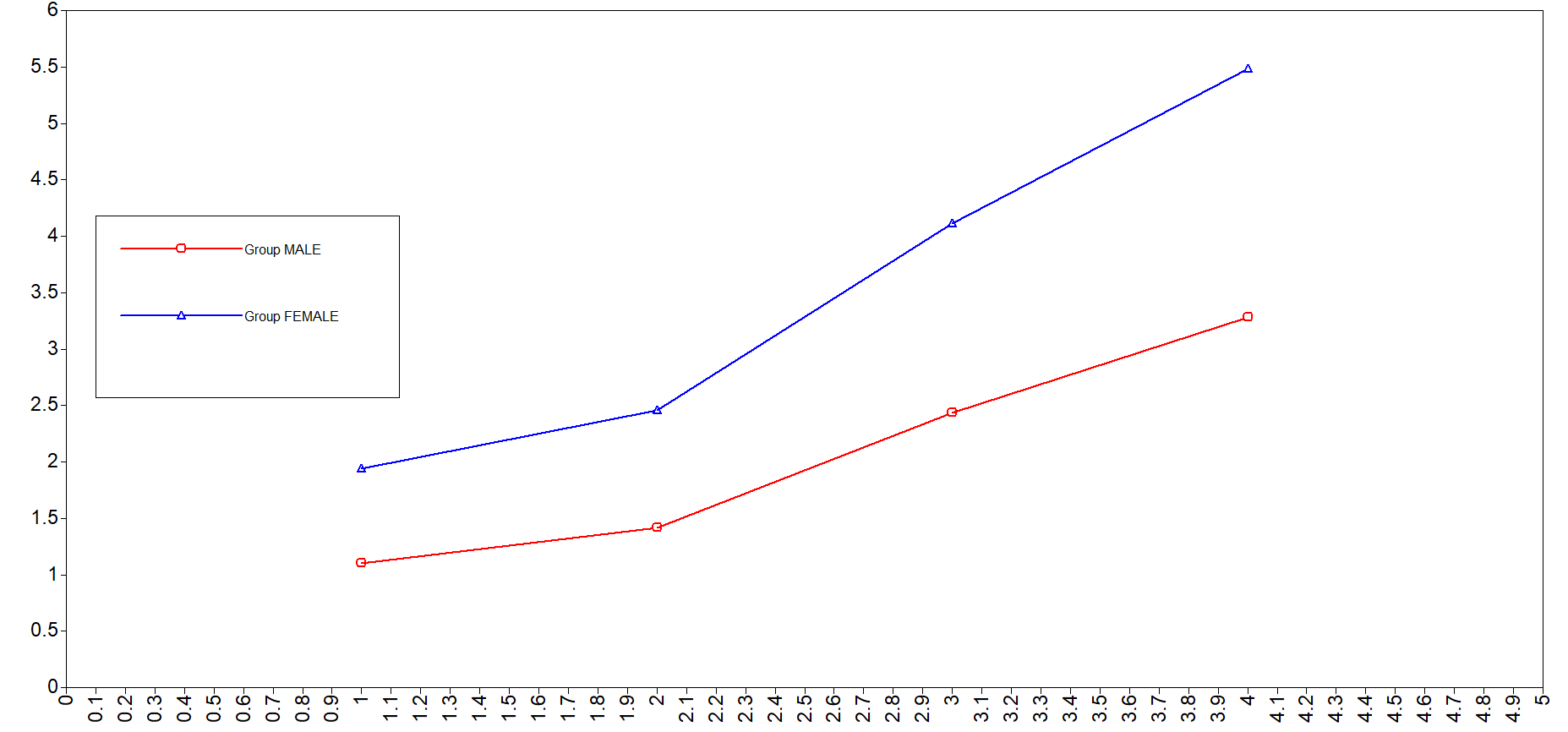
Year

Supplementary Figure 1. Disability Trends of Men and Women Over 11 years of study period.

Supplement: Supplementary file 2 — Additional file 2: Supplementary Figure 1. Disability Trends of Men and Women Over 11 years of study period. [file 12877_2021_2574_MOESM2_ESM.docx]
